# Effects of ultra-fast nanosecond electric pulses on mitochondria transmembrane potential and oxidation

**DOI:** 10.1038/s41598-025-23453-6

**Published:** 2025-11-13

**Authors:** Paulina Malakauskaitė, Augustinas Želvys, Eglė Mickevičiūtė, Veronika Malyško-Ptašinskė, Barbora Lekešytė, Eivina Radzevičiūtė-Valčiukė, Vytautas Kašėta, Vitalij Novickij

**Affiliations:** 1 State Research Institute Centre for Innovative Medicine, Department of Immunology and Bioelectrochemistry, Vilnius, Lithuania; 2 Faculty of Electronics, Vilnius Gediminas Technical University, Vilnius, Lithuania; 3 State Research Institute Centre for Innovative Medicine, Department of Stem Cell Biology, Vilnius, Lithuania; 4 Vilnius Gediminas Technical University, Plytinės g. 27, Vilnius, LT-10105 Lithuania; 5 State Research Institute Centre for Innovative Medicine, Santariskiu g. 5, Vilnius, LT-08406 Lithuania

**Keywords:** Mitochondria, Membrane potential, Calcium electrochemotherapy, Burst compression, High frequency, Permeation and transport, Biological physics, Drug delivery

## Abstract

Electroporation can be successfully employed for controlled molecular delivery and therefore has found clinical applications for treatment of cancer. However, it’s a pulse-dependent phenomenon, thus modulation of the effects is possible by developing new parametric protocols for pulsed electric field generation. In this work, we have developed a generator capable of generating 50 ns pulses with extreme pulse repetition frequency (up to 6.6 MHz), which should enable plasma membrane permeabilization at significantly lower electric field thresholds due to burst compression and modulation of intracellular effects specific to nanosecond range. We have investigated the effects of 6–16 kV/cm, 50 and 300 ns pulses on mitochondria depolarization, followed by ATP depletion study and characterization of mitochondria oxidation. Finally, we have experimentally confirmed the feasibility of the proposed nanosecond pulsed electric field bursts for calcium electrochemotherapy in vitro. For consolidation of knowledge, we have included the results of standard microsecond pulse procedures (8 × 100 µs). As model a CHO-K1-Luc cell line was used. Based on the experimental data, it is concluded that nanosecond pulses (50 ns) when delivered at ultra-fast repetition frequency allow reduction of cell membrane permeabilization thresholds and can be successfully used for calcium electrochemotherapy even with PEF amplitudes as low as 10 kV/cm.

## Introduction

Exposure of biological cells to high-voltage, short-duration pulsed electric fields (PEF) resulting in increase of plasma membrane permeability is known as electroporation^1^. For decades electroporation protocols were dominated by electric pulses from the micro-millisecond duration range^2^. Shorter pulses (e.g., nanosecond) were hardly used since the generation requires specific circuits (i.e., blumleins^3^, which depend on impedance matching and thus have significant limitations for real applications. With the development of semiconductor technology in the past 10 years, the first generators capable of generating sub-microsecond pulses without requirement for impedance matching have appeared^4–6^, thus a new modality of electroporation protocols has been started to be developed. At the same time, based on in silico predictions, it is anticipated that nanosecond pulsed electric field (nsPEF) bursts present a great potential in electroporation applications due to the possibility to affect inner cell structures, including mitochondria^7–9^. Taking into account the crucial role of mitochondria in the complex interactions between cancer and the immune system, the development of treatment strategies to address mitochondria as potential targets is of high priority^10^.

Mitochondria must maintain the electrical potential across their inner membrane within a homeostatic range to sustain normal functioning of the cell^11^. A loss of the proton gradient across the inner mitochondrial membrane often leads to the disruption of the mitochondrial membrane potential (MMP, Δψm) and ATP production^12^. It has been proposed, that mitochondria can lose MMP through opening the mitochondrial permeability transition pores, during PEF^13^. A transient loss of the MMP caused by inner membrane pores may lead to a long-lasting or permanent MMP disruption, which profoundly influences the rate of ATP synthesis^14,15^, which is also linked to calcium overload^16,17^. Calcium, as a second messenger, regulates various cellular responses, however, disrupting mitochondrial calcium homeostasis ultimately causes cell death involving apoptosis or necrosis^18^. Mitochondria are also the main source and target of reactive oxygen species (ROS) in cells^19^, while ROS play an important role in apoptosis induction under both physiologic and pathologic conditions^20^. Recent studies have shown that nsPEFs can modulate ROS in mitochondria and cytosol^21–23^. Although there is no established direct connection between pore formation and ROS, many factors, including Ca^2+^, MMP, and the redox state of mitochondrial components, indicate that ROS influences these processes^13^. As a result, the capability to manipulate the functions of mitochondria by PEF, forms a fundamental base for evolution of electrochemotherapy (ECT) protocols, which are currently dominated by the sequence of 8 × 100 µs delivered at 1 Hz (known as ESOPE protocol)^24^.

Electrochemotherapy (ECT) is a method, which relies on the intracellular drug delivery through electroporation-induced transient pores, therefore, effective diffusion must be ensured to trigger high cytotoxicity^25^. Nevertheless, the conventional nsPEF protocols (delivered at low frequency) have limitations for molecular delivery due to lack of significant electrophoretic component and smaller pore size when compared to microsecond procedures^26,27^. Subsequently, the number of applied works on ECT involving nsPEF is limited and most of the research is focusing cancer ablation, which does not involve application of drugs^28,29^. However, as a solution, the compression of the nsPEF sequence into a high frequency burst (500 + kHz) can be used, which triggers accumulation of residual transmembrane potential throughout the pulse burst significantly boosting drug and gene delivery^30–32^. It was already confirmed both in vitro^31–33^ and in vivo^34,35^, that such ultra-high frequency protocols can be as effective or in some cases even superior to standard ESOPE procedures. For example, previously we have reported that high frequency (1 MHz) bursts when used for calcium ECT trigger a better tumor response, incl. the immunogenic response when compared to ESOPE^35^. While partly the effect can be attributed to the high frequency component of the bursts and thus a more uniform treatment of the tumor due to impedance mitigation^36,37^, however, there is no available data if it could be also influenced by the nsPEF-specific effects on mitochondria.

Previously we have also shown that the permeabilization of the cell plasma membrane is a prerequisite for mitochondria membrane depolarization when PEF in the range of 7.5–12.5 kV/cm and 100 ns pulses were used^15^. Thus, 100 ns (even when delivered at 1 MHz frequency) are not short enough (insufficient frequency component) to ensure selective permeability of mitochondrial membrane. A comparable result can be also achieved by microsecond pulses^38,39^, thus further fundamental research is required to mechanistically understand the effects of high-frequency nsPEF. It should be noted, that there are reports on ultrashort picosecond^40^ or nanosecond^41^ pulses (but low frequency), which induce apoptosis through a mitochondrial-mediated pathway, however, as mentioned above such sequences are hardly applicable for ECT applications. Firstly, such protocols require amplitudes in the range of tens or hundreds of kV/cm, which is technologically challenging in vivo. Secondly, impedance matching is compulsory, which is hard to accomplish in the in vivo or clinical setting. Lastly, the effectiveness of molecular delivery using sub-microsecond, but low repetition frequency burst is inferior to microsecond ECT protocols^42^. Therefore, we believe that compressing the ultra-short pulses into a high frequency burst is currently the most optimal way to reduce the permeabilization thresholds and ensure effective intracellular delivery of target molecules in the context of nsECT.

In this work, we have improved available infrastructure to support 50 ns pulses with more than 6-fold higher repetition frequency (up to 6.6 MHz) to ensure a higher frequency component of the burst and thus speculated if it is sufficient to trigger selective effects on mitochondria without affecting the cell membrane. Additionally, we aimed to derive protocols for 50 ns pulses, which do not require extreme PEF amplitudes (i.e., trigger reduction of permeabilization thresholds), while still ensure excellent treatment efficacy and thus applicability for ECT. We have also characterized the nsPEF-induced (6–16 kV/cm, 50 and 300 ns pulses) mitochondrial oxidation and compared it to ESOPE in the context of calcium electrochemotherapy (CaECT)^43–45^. As it was mentioned above, calcium overload should increase oxidation of mitochondria and ATP depletion, therefore, targeting CaECT in the context of ultrashort pulses is a straightforward choice.

The results have direct application for further in vivo and applied research highlighting the differences between standard and nsPEF-induced ECT. Additionally, this is the first study to characterize the effects of 50 ns and ultra-high pulse repetition frequency (up to 6.6 MHz) pulses for ECT, which was not possible before technologically.

## Results

### Cell membrane permeabilization

**Fig. 1:**
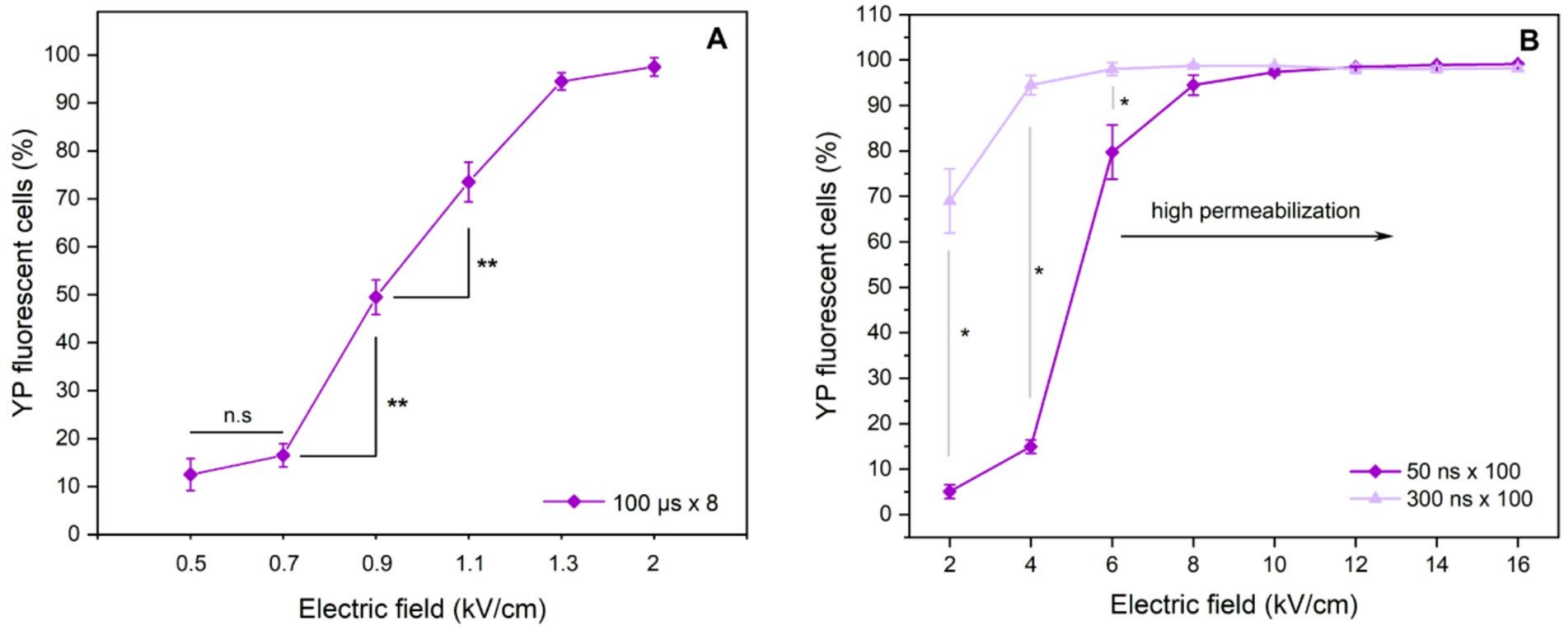
The dependence of cell membrane permeabilization on electric field pulse parameters, where (**A**) 100 µs **×** 8 sequences, 1 Hz repetition frequency; (**B**) 50 ns and 300 ns bursts delivered with a delay of 100 ns (*n* = 100). Untreated control – 6 ± 4% YP-positive cells (data not shown). Asterisks (*p < 0.05; **p < 0.01; n.s – non-significant *p* > 0.05) correspond to differences between highlighted protocols.

To evaluate electroporation efficiency, the cell membrane permeabilization was characterized. The results are presented in Fig. 1. Microsecond protocols returned an expected dose-dependent curve (Fig. 1A). Afterwards, we have analysed the cell membrane permeabilization after application of 50 ns and 300 ns pulses ranging from 2 to 16 kV/cm at a maximum supported pulse repetition rate (delay of 100 ns). The results are summarized in Fig. 1B.

It can be seen that in case of nsPEF, high permeabilization (> 75%) was achieved with pulsed electric field exceeding 6 kV/cm for 50 ns pulses and 4 kV/cm for 300 ns, respectively. As a result, further analysis was limited to 6–12 kV/cm protocols, due to both protocols overlapping in permeabilization efficiency and triggering saturated permeabilization (i.e., the permeabilization rate did not improve with increase of amplitude). The 14–16 kV/cm protocols were not used to ensure reversible permeabilization (refer to viability data further in the paper), which is a pre-requisite for electrochemotherapy. It should be noted that 300 ns pulses, due to longer duration feature a higher energy input (dose), which is delivered to the cells. The comparison of the doses of each protocol used in the study are summarized in Materials and Methods section further in the paper.

### Membrane potential of mitochondria after electroporation

**Fig. 2:**
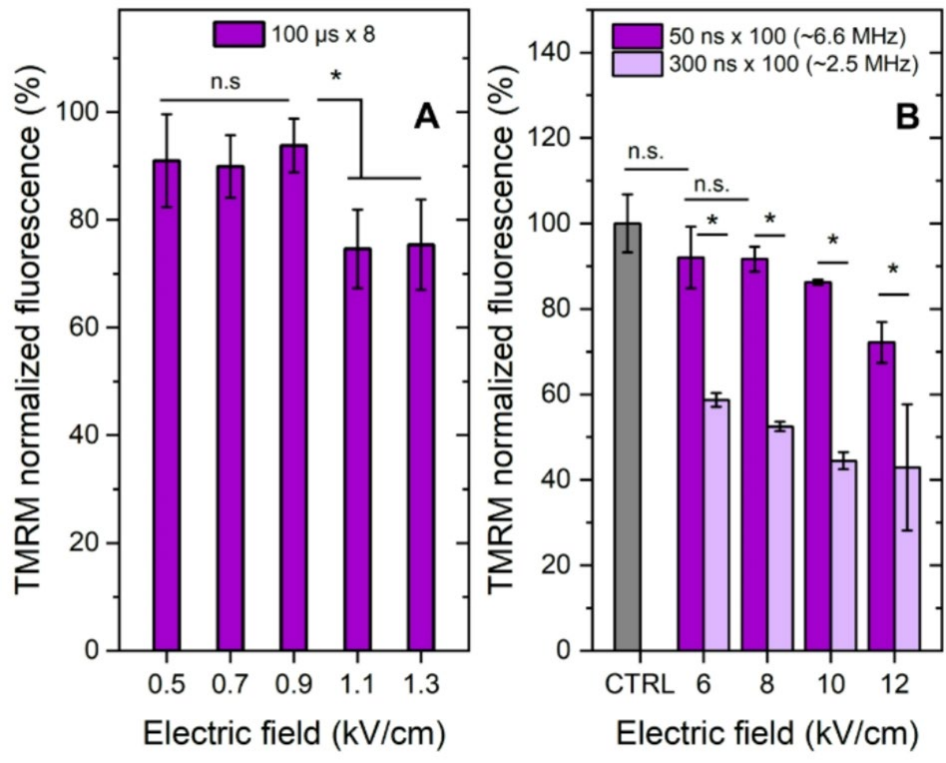
The dependence of MMP in CHO-K1-Luc cells exposed to different pulsed electric field protocols, where **(A)** 100 µs **×** 8 sequences, 1 Hz repetition frequency; **(B)** 50 ns and 300 ns bursts (*n* = 100), delivered with a 100 ns delay between pulses. All data normalized to untreated control (CTRL) and expressed as mean ± standard deviation. Measurements performed 15 min post-treatment. Asterisk (*) corresponds to statistically significant (*P* < 0.05) differences between separate samples.

The changes in the MMP were measured using TMRM dye, which accumulates within the mitochondria in direct proportion to Δψm^46^. The dependences of MMP on applied pulsed electric field are summarized in Fig. 2.

Quantitative fluorescence analysis demonstrated that both µsPEF and nsPEF treatment induced mitochondrial depolarization. After ESOPE pulses (Fig. 2A), only higher amplitudes (1.1 and 1.3 kV/cm) triggered a detectable change in MMP, which indicated that high permeabilization is required to trigger changes in MMP after microsecond pulses (refer to Fig. 1A). In case of nsPEF, a dose dependent response was observed too (Fig. 2B) – the higher was the amplitude, the higher was the depolarization of mitochondria. A distinctive drop in MMP after 300 ns protocols was observed when compared to 50 ns pulses, which is an expected results due to higher energy input. The results again confirmed the hypothesis that high permeabilization of the cell plasma membrane (or in some cases irreversible) was required to trigger significant depolarization of mitochondria.

The application of 50 ns pulses even delivered at extreme frequencies (up to 6.6 MHz) was not sufficient to trigger selective depolarization of mitochondria, i.e., the cell membrane was required to be permeabilized (electroporation). Therefore, based on experimental data it can be seen, that nsPEF (50 + ns) have affected MMP in a similar manner as microsecond pulses (100 µs) – effects were observed only above the electroporation threshold of the cell membrane.

### Oxidative effects following electroporation

**Fig. 3:**
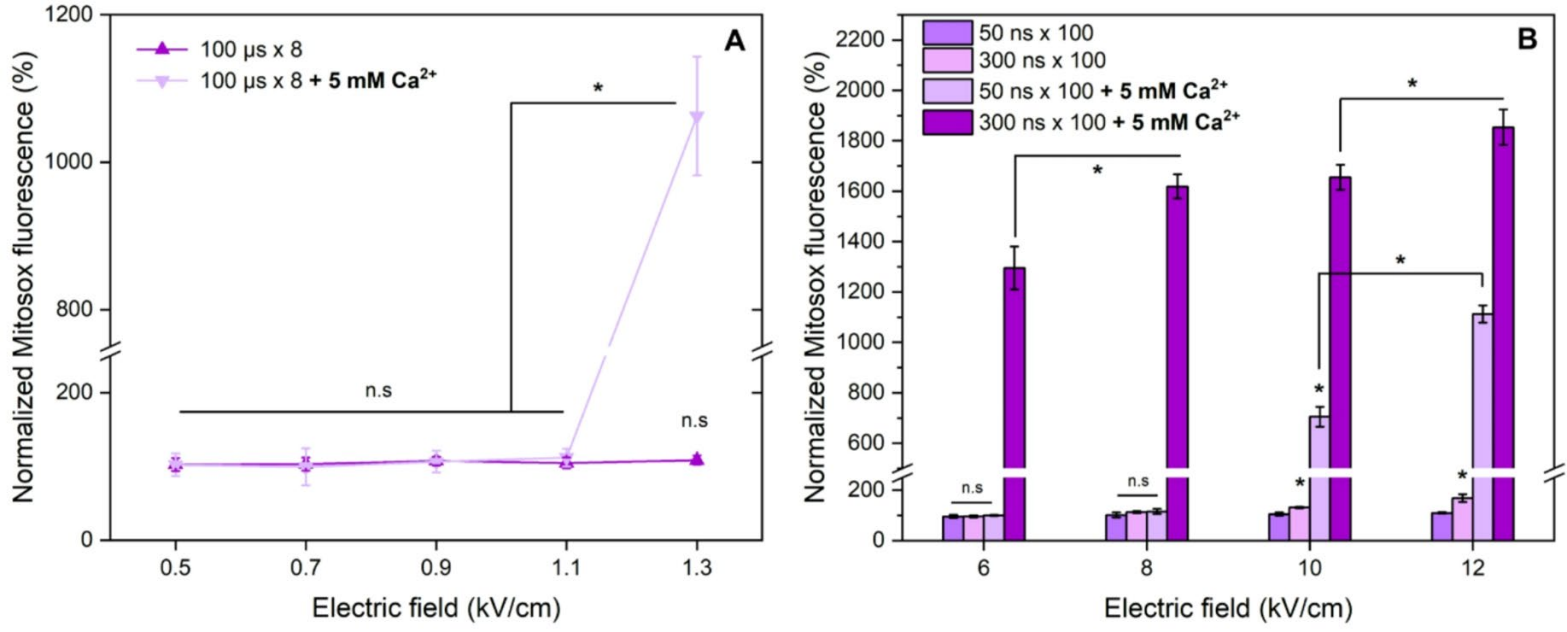
Pulsed electric field induced ROS in mitochondria with and without additional 5 mM Ca^2+^, where (**A**) 100 µs **×** 8 sequences, 1 Hz repetition frequency; (**B**) 50 ns and 300 ns bursts (*n* = 100), delivered with a 100 ns delay between pulses. Measurements performed 15 min post-treatment. Asterisks (*p < 0.05; n.s – non-significant) corresponds to statistically significant differences versus untreated control, unless marked otherwise. All data normalized to untreated control and expressed as mean ± standard deviation.

The same protocols were characterized in the context of mitochondrial oxidation and CaECT (Fig. 3). We have added Ca^2+^ (5 mM, typical dose to study CaECT in vitro^47^.

It can be seen that for ESOPE pulses, the detectable mitochondrial oxidation occurred only in the presence of additional calcium and using the 1.3 kV/cm amplitude, which ensured saturated permeabilization and thus good electrotransfer of calcium ions. The PEF pulses alone did not trigger any detectable change in mitochondrial ROS. However, it was not the case for nsPEF within the studied range of parameters. The 300 ns pulses (10, 12 kV/cm) triggered detectable oxidation of mitochondria (*P* < 0.05 vs. untreated control) even without added calcium, which could be attributed to the higher energy input/dose of the protocols.

In case of 50 ns pulses, the effects were not detectable without calcium, however, when calcium was added, the oxidation increased by more than 700% and 1000% for 10 and 12 kV/cm PEF, respectively. The result is comparable with the ESOPE protocol (1.3 kV/cm). The increase in ROS was even higher (*P* < 0.05, both in respect to ESOPE and 50 ns pulses) when 300 ns pulses (8 + kV/cm) with CaECT were involved, which could be attributed to higher energy input/dose of the protocols and thus, partly irreversible electroporation.

### ATP depletion after Ca^2+^ electrochemotherapy

**Fig. 4:**
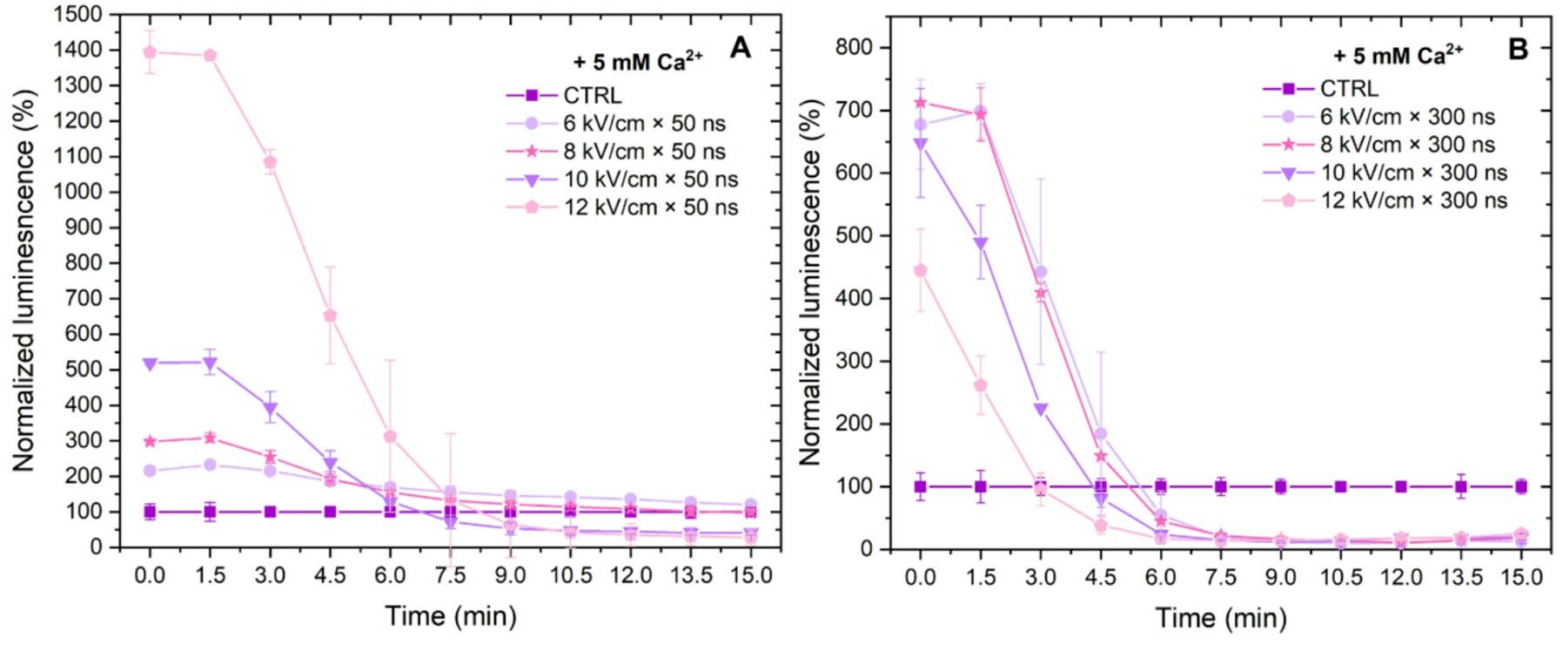
The dependence of ATP depletion on nsPEF-induced Ca^2+^ electrochemotherapy (5 mM), where (**A**) 50 ns and (**B**) 300 ns bursts of 100 pulses, delivered with a 100 ns delay between pulses. All data normalized to PEF-untreated control and expressed as mean ± standard deviation.

The nsPEF protocols involved in our study were also characterized in the context of ATP depletion (proportional to the luminescence of luciferin-luciferase reaction) following Ca^2+^ electrochemotherapy in vitro (Fig. 4). Measurements were conducted at 1.5-min intervals over a total duration of 15 min.

As shown in Fig. 4A, the highest ATP depletion was triggered with the highest PEF amplitude involved in the study (12 kV/cm). The PEF protocols employing 10, 12 kV/cm x 300 ns pulses (Fig. 4B) triggered partly irreversible electroporation or rapid cell death following CaECT (i.e., the cells die instantly after pulse application and only live cells luminesce). In the range of reversible electroporation (e.g., 6 and 8 kV/cm), the signal maximum of the 300 ns bursts was higher when compared to 50 ns protocols, which was an expected result since molecular transfer and membrane damage with longer pulses and higher energy input is expected to be higher.

Due to same reasons, the signal was below the untreated control level sooner for 300 ns pulses when compared to 50 ns protocols (i.e., < 5 min for 300 ns and more than 7 min for 50 ns pulses) indicating cell death. Additionally, since only live cells luminesce, it can be seen that all 6–12 kV/cm protocols resulted in cell death after 7.5 min (no luminescence signal), while for 50 ns protocols only 10 and 12 kV/cm resulted in rapid and effective Ca^2+^ electrochemotherapy. The 6–8 kV/cm did not drop below the untreated control level within the studied time frame, which indicated that molecular transfer was inferior to other protocols involved in the study, and which was also confirmed further in the work by the viability data.

### Cell viability after electroporation and Ca^2+^ electrochemotherapy

**Fig. 5:**
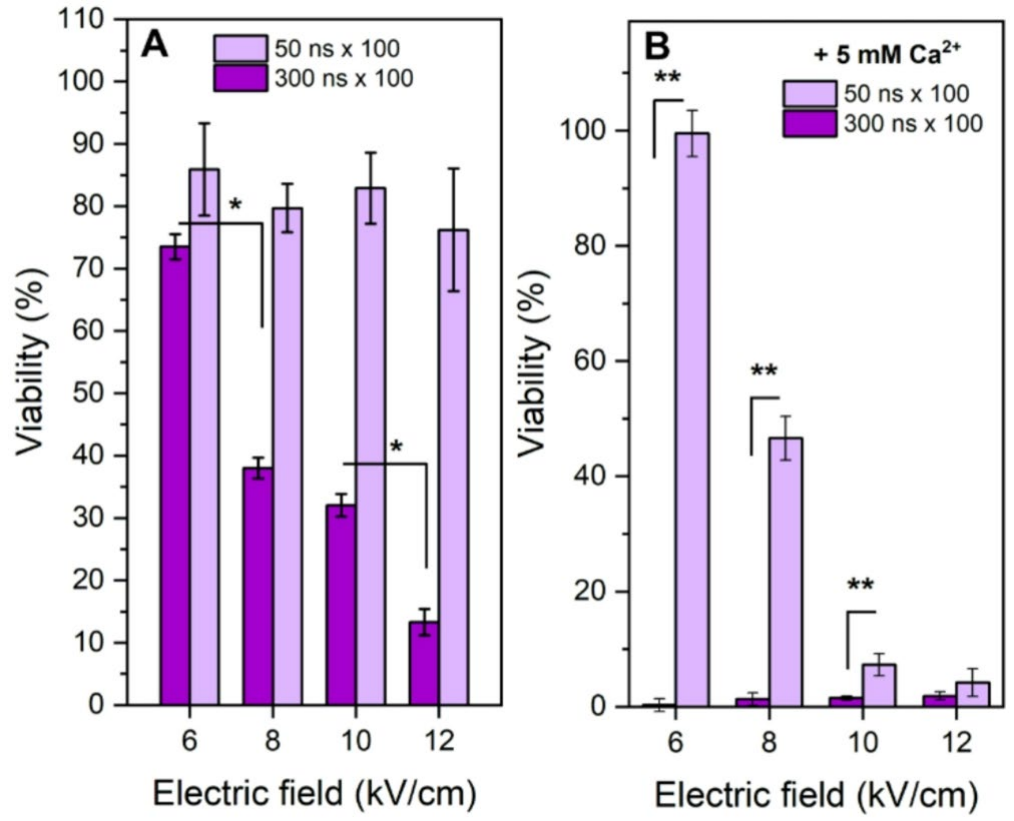
The dependence of CHO-K1-Luc cell viability on PEF parameters without (**A**) and with (**B**) added Ca^2^+ (5 mM). Asterisks (*p < 0.05; **p < 0.01) correspond to statistically significant differences. All data normalized to untreated control and expressed as mean ± standard deviation.

**Fig. 6:**
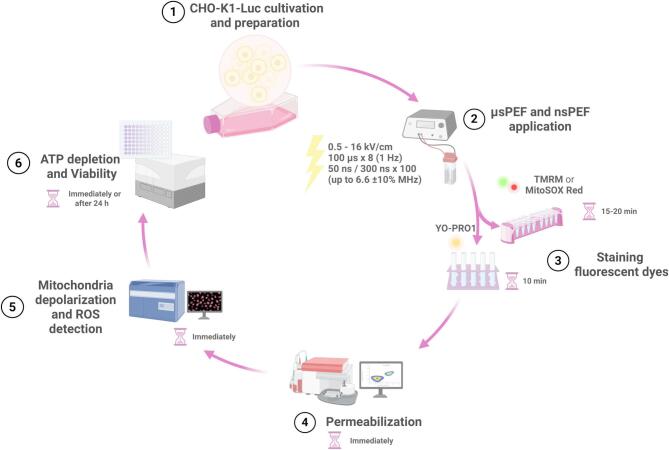
Experimental scheme and methods.

Finally, the viability of the cells after nsPEF and CaECT treatment was evaluated 24 h post-treatment and is presented in Fig. 5. It can be seen that 300 ns pulses triggered irreversible or partly irreversible electroporation in the 8–12 kV/cm range (which was predicted by ATP depletion data, Fig. 6), while 6 kV/cm protocol can be used for electrochemotherapy (viability ~ 75%).

The 50 ns pulses did not trigger any statistically significant loss in viability independently on the used amplitude when there was no added calcium. However, when combined with CaECT the 10 and 12 kV/cm protocols successfully induced ESOPE-equivalent cytotoxicity (4 ± 3% (data not shown) at 1.3 kV/cm PEF), which is in agreement with ATP depletion data (refer to Fig. 4).

Naturally, when used in the CaECT context, the 300 ns pulses killed all the cells independently on the protocol (Fig. 5B), since most of the protocols (i.e., 8–12 kV/cm) triggered predominantly irreversible electroporation by PEF alone.

## Discussion & conclusions

A hypothesis, supported by in silico data, posits that nsPEF can induce selective permeabilization of intracellular structures, such as mitochondria, without affecting the integrity of the membrane^48,49^. However, one of the major findings in our previous research is that 100 ns even when delivered with 1 MHz repetition frequency, were not suitable to trigger selective permeabilization of mitochondria and permeabilization of the cell membrane was still a pre-requisite^15^. At the same time, it was shown that high frequency 100 ns pulses still can be used for the calcium electrochemotherapy, but the input energy required to trigger high permeabilization with pulses up to 12.5 kV/cm should be several-fold higher than the one required for ESOPE protocols^15^. Such a result significantly hinders the potential applicability and benefits of nsPEF for ECT. Application of high amplitude electric field pulses is already challenging in a clinical context requiring generators supporting tens of kV, therefore, going even higher in terms of PEF amplitude will produce even more engineering problems for isolation, voltage breakdown prevention and safety. Therefore, in our opinion, the most relevant solution is to further reduce the pulse duration and increase the pulse repetition rate, without dramatic increase in pulse amplitude, which was implemented in this work. As a result, the decision to increase the repetition frequency of the pulses (up to 6.6 MHz) due to residual TMP accumulation phenomenon^50^ enabled saturated cell membrane permeabilization already at 8 + kV/cm using pulses as short as 50 ns. Normally amplitudes in the range of tenths of kV/cm are required for such durations (when lower frequency bursts are involved)^51^. Nevertheless, it was still not-sufficient to cause detectable selective effects on MMP. Therefore, we conclude that pulses shorter than 50 ns with further increase of repetition frequency are required. In our opinion, increase of the repetition frequency is inevitable, since low frequency bursts will require high PEF amplitudes, which is hardly applicable for real ECT applications.

Additionally, we characterized the mitochondrial oxidation following nsPEF pulses and CaECT. The mitochondrial oxidation is higher with nsPEF pulses causing irreversible electroporation when compared to ESOPE procedure. The nsPEF alone (i.e., the 300 ns bursts of 8 + kV/cm) due to high energy input and partly irreversible electroporation can trigger sufficient intracellular ROS to oxidize mitochondria by PEF alone, while the oxidation increases by several orders of magnitude when calcium is added. The result is in agreement with established knowledge that calcium overload induces ROS in mitochondria^52^. It should be also noted that the effects are not a by-product of electrolysis, which is typical for micro-millisecond pulses^53^. The electrolysis products of nsPEF are significantly lower than the ones produced by longer pulses^42^. In this work, only 100 pulses in a burst were used, which is only a fraction of those (i.e., *n* = 1000) involved in previous works^15^. Now we were able to reduce the PEF amplitude to 10 kV/cm, reduce the duration to 50 ns and 10-fold reduce the number of pulses, but still ensure excellent cytotoxicity of nsPEF ECT, which was possible only due to burst compression and extreme repetition frequency of 6.6 MHz. Finally, we have tested the proposed protocols in the context of CaECT in vitro. It was expected that shorter pulses may result in lower molecular transfer^42^, therefore, we have used both 50 and 300 ns pulses throughout the work. CaECT relies on calcium influx, resulting in rapid ATP depletion followed by cell death^54^. Calcium influx also dramatically influences both the cell viability and oxidation effects. During cell death, mitochondrial dysfunction leads to electron leakage and an impaired ability to regulate ROS levels, resulting in excessive ROS accumulation^55^. Our results indicate that successful intracellular calcium delivery increases oxidation of mitochondria by several orders of magnitude and the result can be triggered both by 50 ns and 300 ns pulses when they are compressed into a high frequency burst, which in the future can be utilized for modulation of cell death type^56^.

We have also characterized the kinetics of ATP depletion following the treatment. As expected, both the 50 and 300 ns protocols can result in saturated cytotoxic effect, however, pulses with higher energy input are more effective for molecular delivery, which is in agreement with established theory^50^. Even the predominantly reversible electroporation range, the cells die (no luminescence signal) in less than 5 min with 300 ns pulses, while it takes more time (i.e., >7 min for 50 ns pulses) highlighting differences in passive diffusion between the nsPEF protocols. Due to differences is energy input/dose and the time the cells are exposed to PEF, the 300 ns pulses can be successfully used for CaECT already at 6 kV/cm, while the PEF amplitude of 10 + kV/cm is required for 50 ns pulses (within the studied range of parameters). In the clinical context, lower amplitude is beneficial due to engineering and safety reasons, however, higher frequency component of the bursts is beneficial in terms of impedance mitigation and more uniform electric field distribution within the tumor^36^.

To summarize, it is the first work to characterize the effects of ultra-high frequency pulses (>2 MHz) as short as 50 and 300 ns for application in the context of CaECT. It is concluded, that nsPEF can be used for ECT even with low PEF amplitude (< 10 kV/cm), while potentially bringing the benefits for impedance mitigation and more uniform electric field distribution within the tumor. However, the selective permeabilization of mitochondria is still unachievable even when 50 ns pulses are used. The permeabilization of the cell plasma membrane is still a pre-requisite for mitochondrial depolarization, which is similar to microsecond bursts^38^. Currently, we are at the limit of available semiconductor technology, therefore, going significantly higher in terms of pulse repetition frequency and further shortening the pulse duration requires development of new transistors and/or scheme topologies. Nevertheless, the effects and efficacy rates of ECT reported in our work are already promising since allow to trigger high cytotoxicity with mid-range PEF, while utilizing extremely short pulses (i.e., 50 ns). The effects should be further justified in the in vivo context since the heterogeneity of tissue and challenges in ensuring electric field homogeneity could be highlighted as the major limitation of microsecond protocols, while the proposed parametric protocols potentially solve the problem by impedance mitigation in the high frequency range.

## Materials and methods

In this work, a new modality of ultra-high frequency (up to 6.6 MHz) nanosecond protocols (50 ns and 300 ns) was used to characterize the differences on bioeffects between nsPEF and ESOPE protocol. As a cell model, CHO-K1-Luc cells were used and the membrane permeabilization, mitochondria membrane potential (MMP) changes, ROS, and viability separately and in the context of CaECT were evaluated. For mitochondrial effects characterization, two different fluorescent probes were used: TMRM and MitoSOX Red for MMP and ROS detection, respectively. For permeabilization of cell plasma membrane characterization the Yo-Pro-1 was used. Additionally, the CHO-K1-Luc cell line enabled the characterization of ATP depletion kinetics following the treatment based on luciferin oxidation reaction intensity^57,58^. The summarized experimental scheme is shown in Fig. 6.

Each procedure is described in detail further in the paper.

### Pulsed power setup and protocols

The electric pulses were applied using up to 3 kV square-wave high-voltage pulse generator (VilniusTECH, Vilnius, Lithuania) capable of generating bursts of pulses at a pre-defined frequency within the range of 1 Hz to 5 MHz^59^. In order to generate shorter pulses, the generator was adapted to the experiment by switching to faster C2M0160120D (Wolfspeed, Durham, USA) MOSFETs instead of using the original ones, which enabled generation of ~ 50 ns pulses with ~ 100 ns delay between them.

**Fig. 7:**
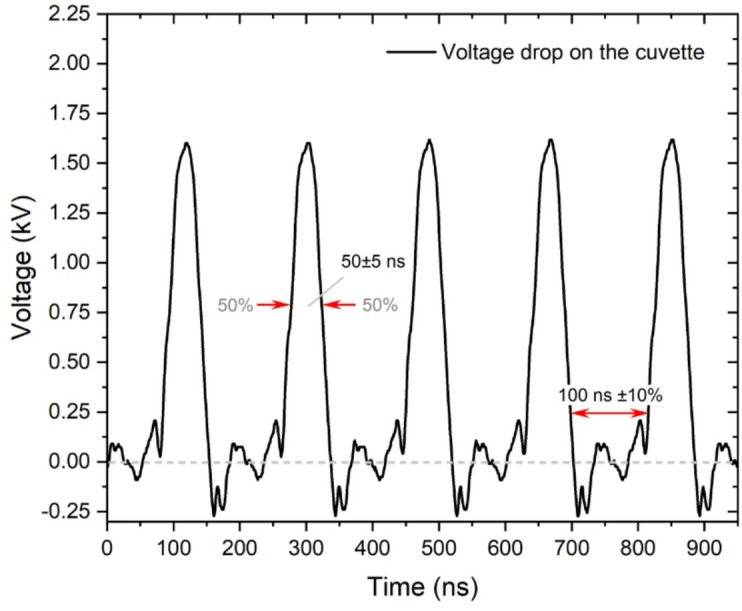
The measured waveform (directly on the cuvette) of the ~ 50 ns pulses. The waveform was acquired using a TAO3000 oscilloscope (Owon Technology, Ontario, Canada) and a 1:100 P4250 probe (Cleqee, Shenzhen, China).

The electroporation cuvette with 1 mm gap between parallel plate aluminium electrodes (VWR International, USA) was used as a load. The applied voltage varied in 50 V – 1.6 kV range, corresponding to a 0.5–16 kV/cm electric field in the cuvette. The 50 ns and 300 ns monophasic pulses were delivered with a delay of 100 ns between the pulses, with a total number of 100 pulses in a burst. The microsecond pulses (0.5–2 kV/cm x 100 µs x 8 sequences, 1 Hz) were used as reference protocols for incorporation and comparison of knew knowledge and data on nsPEF. The shortest possible pulse waveform is shown in in Fig. 7.

The summary of the pulse protocols involved in the study including the delivered dose^60^, which is proportional to delivered energy are summarized in Table 1.

**Table 1:** The summary of the pulse protocols involved in the study.

Electric field [kV/cm]	Duration [µs]	Number of pulses, *n*	Dose (U^2^ × t) [V^2^ × s]
Microsecond protocols			
0.5	100	8	2.0
0.7	100	8	3.9
0.9	100	8	6.5
1.1	100	8	9.7
1.3	100	8	13.5
2	100	8	32.0
Nanosecond protocols			
2	0.05	100	0.2
2	0.3	100	1.2
4	0.05	100	0.8
4	0.3	100	4.8
6	0.05	100	1.8
6	0.3	100	10.8
8	0.05	100	3.2
8	0.3	100	19.2
10	0.05	100	5.0
10	0.3	100	30.0
12	0.05	100	7.2
12	0.3	100	43.2
14	0.05	100	9.8
14	0.3	100	58.8
16	0.05	100	12.8
16	0.3	100	76.8

### Cell culture

The CHO-K1-Luc cell line was developed (long-term electrotransfected using linearized pcDNA3.1(+)/Luc2 = tdT plasmid (CHO-Luc) in State Research Institute for Innovative Medicine (Vilnius, Lithuania)^61^. Cell culture was maintained in RPMI 1640 culture medium supplemented with 10% (y volume) fetal bovine serum (FBS) (Gibco, Thermo Fisher Scientific, USA) and 1% (by volume) penicillin/streptomycin. Cells were sustained in humidified incubators at 37 °C and 5% CO_2_ and all experiments were performed at 80% cell culture confluency.

On the experimental day CHO-K1-Luc were washed twice with PBS (Gibco^®^, Waltham, MA, USA) detached using trypsin (Thermo Fisher Scientific, Waltham, MA, USA), centrifuged, and resuspended in HEPES buffer (10 mM HEPES (C8H18N2O4S, Sigma-Aldrich, cat. no.: H337) with sucrose and 1 mM Mg^2+^) in different concentrations depending on the experiment.

### Permeabilization detection assay

Electroporation-induced cell permeabilization in CHO-K1-Luc cells was identified using the green fluorescent dye Yo-Pro-1 (YP, Sigma–Aldrich, St. Louis, MO, USA). Cells at 2 × 10^6^/ml concentration and resuspended in HEPES buffer were mixed with YP dye to achieve a final concentration of 1 µM (YP). The 60 µL samples were placed inside the cuvette between the electrodes and treated with microsecond or nanosecond electroporation protocols. Afterwards, the cells were transferred into a 96-well round-bottom plate (Nunc, Sigma–Aldrich, St. Louis, MO, United States of America). Following a 10 min incubation at room temperature, 150 µL of 0.9% NaCl (Chempur, Piekary Slaskie, Poland) solution was added. The control samples without treatment were used as a negative control for gate definition. After incubation, samples were measured using a BD Accuri C6 flow cytometer (BD Biosciences, San Jose, CA, United States of America), where YP (Ex. 491⁄509) fluorescence was detected in Channel FL1 (Em. 533/30 nm band-pass filter). Results analysis performed with BD Accuri C6 Software (BD, Franklin Lakes, NJ, USA) and later post-processed in OriginPro software (OriginLab, Northampton, MA, USA).

### Mitochondrial membrane potential characterization

MMP was detected with tetramethylrhodamine methyl ester (TMRM; T668; Thermo Fisher Scientific, Landsmeer, Netherlands). It is a cell-permeant, cationic dye that accumulates in active mitochondria with intact membrane potentials and produces bright red-orange fluorescence^62^. On the experiment day, cells were centrifuged, resuspended in HEPES buffer (6 × 10^6^/ml) and incubated on ice for 20 min. The 60 µl of samples were placed between the electrodes and treated with different PEF protocols. After the treatment the cells were stained with 33 nM of TMRM and incubated at 37 °C for 15 min. Changes in MMP using TMRM (Ex. 552/574) were detected and analyzed with flow cytometer FlowSight (Amnis, Seattle, WA, USA) using 488 nm laser and 577/35 band-pass filter. Data on median fluorescence of individual cells (1 × 10^4^) were normalized to untreated control and used for further analysis.

### Determination of mitochondrial oxidation

The ROS levels were determined using MitoSOX Red (Thermo Fisher Scientific, Waltham, MA, USA), a red fluorogenic dye that is selectively targeted by mitochondrial superoxide, but not by other ROS generating systems^63^. Cells were centrifuged, resuspended in HEPES buffer (7 × 10^6^/ml) and incubated on ice for 20 min. Before the PEF treatment, additional CaCl_2_ (final concentration of 5 mM) was either added or not, depending on the protocol. The 60 µl samples were placed between the electrodes and treated with different PEF protocols. After PEF application the cells were stained for 20 min containing 5 µM of MitoSOX at 37 °C. Mitochondrial ROS was detected and analyzed with flow cytometer FlowSight (Amnis, Seattle, WA, USA) using 488 nm laser and a 577/35 band-pass filter. Data on median fluorescence of individual cells (1 × 10^4^) were normalized to untreated control and used for further analysis.

### Determination of ATP depletion

The prepared cell suspension (1 × 10^6^/ml) after 20 min incubation on ice was treated with different PEF protocols with additional CaCl_2_ (5 mM). After treatment the samples were instantly transferred to white 96-well flat bottom plate (Thermo Fisher Scientific, Waltham, MA, USA). D-Luciferin (Promega, USA) was added to the samples at final concentration of 150 µg/ml. ATP depletion was assessed using D-Luciferin (Promega, USA) oxidation reaction^64^. Immediately, the luminescence was measured for up to 15 min with a 1.5 min time step. The luminescence was evaluated using a Synergy 2 microplate reader and Gen5 software (BioTek, USA).

### Electrochemotherapy and viability assay

The cells viability 24 h post-treatment was characterized based on PrestoBlue (Thermo Fisher Scientific, Waltham, MA, USA) metabolic activity assay (Thermo Fisher Scientific, Waltham, MA, USA). Prepared cell suspension (1 × 10^6^/ml) was incubated on ice and later treated by different PEF protocols (with or without additional CaCl_2_ (5 mM)). Later the samples were transferred to a 96-well flat bottom plate (TPP, Trasadingen, Switzerland) and incubated in room temperature. After 10 min the growth medium was added to each well and incubated at 37 °C and 5% CO_2_ for 24 h. The following day, the wells underwent three washes with PBS. Afterwards, 150 µl of PBS and 5 µl of cell viability reagent PrestoBlue, were added to each well. The cells were incubated for the next 2 h at 37 °C, 5% CO_2_ and the fluorescence was measured using a Synergy 2 microplate reader and Gen5 software (BioTek, Shoreline, WA, USA). The excitation wavelength was 540/20 nm and the emission was evaluated at 620/40 nm. All the data were normalized to untreated control.

### Statistical analysis

One-way analysis of variance (ANOVA; *p* < 0.05) was used to compare different treatments. Tukey HSD multiple comparison test for the evaluation of the difference was used when ANOVA indicated a statistically significant result (*p < 0.05* was considered statistically significant). The data was post-processed in OriginPro software (OriginLab, Northampton, MA, USA). All experiments were performed at least in three repetitions, and the treatment efficiency was expressed as mean ± standard deviation.

## Data Availability

Data available on request from the corresponding author(PM or VN)**.**
